# A conceptual framework for psychiatric nurses to facilitate medication compliance among adults living with depression

**DOI:** 10.4102/curationis.v47i1.2581

**Published:** 2024-10-04

**Authors:** Jeanne du Plessis, Annie Temane, Marie Poggenpoel

**Affiliations:** 1 Department of Nursing, Faculty of Health Sciences, University of Johannesburg, Doornfontein, South Africa; 2 Department of Nursing, Faculty of Health Sciences, University of Johannesburg, Johannesburg, South Africa

**Keywords:** conceptual framework, internal locus of control, medication compliance, adults living with depression, non-compliance with medication

## Abstract

**Background:**

Medication non-compliance is a significant healthcare issue that is widespread. Extensive research has identified factors that contribute to medication non-compliance in different healthcare settings. As a result, there was a need to develop a conceptual framework to facilitate medication compliance among adults living with depression.

**Objectives:**

The purpose of this study was to develop a conceptual framework for psychiatric nurses to facilitate medication compliance among adults living with depression.

**Method:**

A qualitative, exploratory, descriptive and contextual research design was utilised to investigate the experiences of adults living with depression who are non-compliant with medication. The study consisted of three phases: an empirical phase, a classification of concepts and a development phase. Following the empirical phase, a conceptual framework was developed based on the classified concepts.

**Results:**

Three sets of results were merged for the cross-validation analysis, combining findings from the systematic review, the researcher’s previously conducted and published minor dissertation and the current study.

**Conclusion:**

A conceptual framework was developed to assist psychiatric nurses in effectively promoting an internal locus of control among adults living with depression. The resulting conceptual framework provides valuable insights and serves as a valuable tool for future research endeavours aimed at enhancing medication compliance among adults living with depression.

**Contribution:**

This framework serves as a valuable guide for future studies that aim to explore medication compliance among adults living with depression, specifically by focussing on the concept of internal locus of control.

## Introduction

Depressive disorders are common mental health conditions that are estimated to affect approximately 280 million people worldwide (WHO 2023:n.p.). Recent research conducted by Vilsaint et al. (2019:53) suggests that depressive disorders are more prevalent than previously thought, affecting 16.5% of the global population. Recent data from a South African national population survey conducted by Masemola, Moodley and Shirinde (2022:n.p.) reported that 27.1% of South Africans exhibited symptoms of depressive disorder. A comprehensive nationwide study discovered that 20.9% of men and 26.4% of women experienced severe symptoms of depressive disorder (Gibbs, Govender & Jewkes 2018:789). While data are scarce regarding the epidemiology of major depressive disorders in South Africa, studies conducted between 2005 and 2015 revealed a staggering 41.0% increase in the occurrence of mental disorders (Bateman 2014:7).

Depressive disorders are frequently associated with severe and recurring symptoms that can lead to significant psychosocial impairment and increased mortality (Tehranchi et al. 2018:1609). According to the Diagnostic and Statistical Manual of Mental Disorders, Fifth edition (DSM-5), depressive disorders represent a group of disorders including disruptive mood dysregulation disorder, major depressive disorder, persistent depressive disorder, premenstrual dysphoric disorder, substance and/or medication-induced depressive disorder, depressive disorder because of another medical condition, and other specified and unspecified depressive disorders (American Psychiatric Association 2013). These depressive disorders are commonly characterised by episodes of persistent feelings of sadness, irritability and a loss of interest or pleasure in daily activities for a minimum of 2 weeks. These episodes can greatly affect an individual’s emotional, cognitive and physical well-being, resulting in a decrease in overall functioning and quality of life (American Psychiatric Association 2013). Depressive episodes must occur for a period of 2 weeks in order to establish a diagnosis (American Psychiatric Association 2013; Benazzi 2006:n.p.).

Furthermore, it is worth noting that depressive disorders often coexist with other mental disorders, particularly anxiety and substance use disorders (Kalin 2020:366). As a result, individuals experiencing depressive disorders are likely to face significant impairments in their performance, leading to substantial personal and socioeconomic challenges (Amanat et al. 2022:676). Moreover, a recent analysis of medical insurance data revealed a concerning trend of a significant number of adults suffering from depressive disorder but not receiving the appropriate treatment in accordance with established guidelines (Steffen et al. 2020:240). Against this backdrop, it becomes imperative to prioritise improved care for individuals battling depressive disorder. Understanding the experiences associated with this mental disorder is critical, particularly given the growing number of afflicted individuals in South Africa and worldwide. Studies have indicated a significant correlation between locus of control and mental health.

Locus of control is described as the individuals’ belief that events occur as a result of their own actions and behaviours (internal locus of control) or by powers outside of themselves such as a divine entity (external control) (Atiri 2018:254; Lopez-Garrido 2020:n.p.; Rothmann 2001:45). An internal locus of control is a way to construct a life of meaning with purpose, significance, fulfilment and satisfaction (Hussain et al. 2020:13). An internal locus of control is actualised through positive functioning, understanding the importance of medication compliance, happiness, positive affect and hope (Poalses 2022:38). Having an internal locus of control entails believing that one’s actions and decisions directly impact life’s outcomes (Main 2023:n.p.). In contrast, adults living with depression without an internal locus of control tend to attribute their failure to comply with medication to external factors (Zawawi & Hamaideh 2009:75). Adults living with depression may feel helpless and powerless, leading to a lack of motivation and a sense of resignation. This can result in missed opportunities and a failure to achieve personal goals. Higher levels of internal locus of control are associated with lower levels of depression and higher levels of external locus of control referring to both powerful others and chance would be associated with higher levels of depression (Khumalo & Plattner 2019:2; Main 2023:n.p.).

Non-compliance with psychiatric medication can pose significant challenges for individuals who rely on an external locus of control (Myers 2020:n.p.); yet, depressive disorders have been identified as a contributing factor that may lead to poor compliance with medication (Acharya & Agius 2018:631). A recent meta-analysis study conducted by Solmi et al. (2020:190) revealed that individuals with depressive disorders were three times less likely to comply with medication regimens compared to those with other conditions. Furthermore, research conducted in the United States (US) found that 4.8% of patients did not initiate antidepressant treatment in the first 6 months after receiving a prescription, while 12.2% discontinued treatment within the first 6 months (Chawa et al. 2020:n.p.). Kim et al. (2018:747) concur that compliance with prescription medication for serious mental disorders significantly declines after 6 months of therapy. In support, Naghavi et al. (2019:2) highlight that between one-third and half of long-term prescribed psychiatric medications are not used as directed, and in the case of depressive disorders, approximately 76.0% of patients do not comply with their prescribed medication.

One of the primary reasons individuals choose not to continue taking antidepressants relates to their anxiety regarding potential adverse drug reactions. Several other factors contribute to the early discontinuation of antidepressant medications, including the severity of the condition, comorbidity, personality traits and insufficient support from healthcare providers (Solmi et al. 2020:190). However, the most significant factor leading to the termination of antidepressant treatment is the lack of effectiveness in alleviating symptoms (Kelly, Posternak & Jonathan 2022:n.p.).

Compliance can be defined as patients’ successful, voluntary and reciprocal participation in an appropriate course of action to achieve a therapeutic outcome (Kvæl et al. 2019:n.p.). Specifically, medication compliance refers to patients adhering to prescribed medicines as directed by their doctors and following ongoing instructions throughout psychiatric drug therapy (Kvarnström, Airaksinen & Liira 2018:5). Recent research has emphasised that ‘compliance’ is a term that encompasses patients’ willingness to be treated and their understanding of the necessity for such treatment. It reflects patients’ level of tolerance and comprehension of the medications prescribed to them, and whether they approach it with a supportive or negative mindset (De las Cuevas & De Leon 2020:1832).

Medication compliance is ultimately a global concern for healthcare professionals, the healthcare system, family members, caregivers and other stakeholders. Extensive evidence suggests that non-compliance is widespread and linked to unfavourable outcomes and increased healthcare costs (Semahegn et al. 2018:2). According to Malik, Kumari and Manalai (2020:25), one of the major challenges associated with non-compliance is increased morbidity and mortality rates. However, if non-compliance issues can be identified and addressed, it has the potential to significantly improve patients’ mental health, reduce the burden on patients, prevent relapse and enhance the overall quality of mental healthcare services (Samartzis & Talias 2020:249).

Compliance with psychiatric medication is thus a significant concern in the treatment of depressive disorders. Studies have also demonstrated personal experiences with antidepressant medications have implications for adherence to therapy when these medications are discontinued. Despite the widespread use of psychiatric medications as a vital tool in treating depressive disorders, data from comprehensive epidemiological research indicates that only one in three patients successfully complete medication therapy (Burke et al. 2019:326). Non-compliance with treatment among different classes of psychiatric patients is thus a growing problem that can lead to treatment failure and cause serious challenges for patients.

Furthermore, individuals suffering from severe mental disorders who fail to comply with their prescribed medications can inadvertently worsen their condition. This can lead to a range of complications, including re-hospitalisation, adverse psychosocial outcomes, exacerbated symptoms, reduced efficacy of subsequent therapies, improper use of limited healthcare resources, heightened substance abuse, diminished quality of life and an elevated risk of suicide (Williams, Jones and Reddon 2019:n.p.). Conversely, patients’ optimistic attitudes and active engagement with their healthcare providers play a pivotal role in determining their compliance with antidepressant medications (Tickell et al. 2020:n.p.).

The role of the psychiatric nurse is to provide care, treatment and rehabilitation services to the mental healthcare user (*Mental Health Care Act 17 2002*:n.p.). Furthermore, the psychiatric nurses’ role in this research study is to identify gaps and to contribute to practice by implementing research (SANC 2018:2). Psychiatric nurses play a crucial role as the primary point of contact for patients, acting as a bridge between complex medical terminology and patient comprehension. They are tasked with educating patients on their medications, emphasising the importance of adhering to prescribed regimens, potential side effects and the repercussions of non-compliance. By nurturing therapeutic relationships, psychiatric nurses facilitate open communication, creating a safe space for patients to express concerns, fears or misunderstandings about their treatment plans.

A psychiatric nurse in South Africa is a professional nurse with a basic 4-year nursing qualification registered with the South African Nursing Council (SANC) under R425 of 1985 (as amended by R753 of 1988) and an advanced psychiatric nurse holds an additional qualification in psychiatric nursing sciences in accordance with the *Mental Health Care Act No.17 of 2002* (*Mental Health Care Act 17 2002*:n.p.; Regulation 425 1985:n.p.). Furthermore, the advanced psychiatric nurse is equipped with advanced mental health competencies. The advanced psychiatric nurse facilitates the psychiatric nurses by assisting them in mentoring, supervising, guiding and providing support for the psychiatric nurse in facilitating adults with depression to develop an internal locus of control.

The primary objective of this study was to develop a conceptual framework that effectively enhances medication compliance among adults living with depression by using an internal locus of control. A conceptual framework can be described as a tool that comprehensively explains the main components to be studied, the key factors, concepts or variables, and the presumed relationships among them. According to Polit and Beck (2018:192), a conceptual framework outlines strategic objectives in conjunction with an action plan to achieve the research aim. Essentially, it is a guiding principle that directs researcher towards realising their studies’ objectives. It represents researchers’ synthesis of existing literature on how to explain a phenomenon. A conceptual framework for psychiatric nurses to facilitate medication compliance among adults living with depression will guide psychiatric nurses as they work towards empowering adults living with depression to develop an internal locus of control. The development of a comprehensive framework was essential to assist nurses in ensuring medication compliance, ultimately enhancing therapeutic outcomes and streamlining the medication administration process in healthcare settings.

### Problem statement

There is a significant lack of support for adults living with depression who do not comply with their medication. This phenomenon reflects the pressing need to develop a comprehensive conceptual framework to support these individuals sufficiently. By establishing a conceptual framework specifically designed for non-compliant adults living with depression, the focus can be shifted towards addressing the multitude of challenges they face rather than merely tackling one issue. This approach will result in minimal interventions while effectively improving medication compliance among adults living with depression, empowering them with an internal locus of control. For this framework to be deemed effective, it must address the challenges that these individuals encounter, as these challenges have a detrimental impact on their overall well-being.

### Purpose of the study

The purpose of this study was to develop a conceptual framework to support adults living with depression and struggling with medication compliance.

## Research methods and design

This research project utilised a three-phase method. The first phase involved an empirical approach consisting of two stages: a systematic review and qualitative research methods. These methods included an exploratory, descriptive and contextual research design to gain insight into the experiences of adults living with depression who do not comply with psychiatric medication. The study drew upon the works of Creswell & Poth (2018:292), Gray and Grove (2021:247–313) and Duda, Warburton and Black (2020:33–34) to inform the research design and methodology. The second phase was the classification of the central concept phase. The third phase was the development of the conceptual framework to facilitate medication compliance among adults living with depression using an internal locus of control.

The results of the researchers’ previous minor dissertation were used (du Plessis et al. 2021:1). A qualitative, exploratory, descriptive and contextual research design was utilised, embracing a phenomenological approach. Family members aged between 20 and 45 years were purposively sampled. Data were collected through eight in-depth phenomenological interviews. Open-ended questions were asked. The central question asked was:

Tell me your experiences of living with your wife, mother, sister and daughter living with depression and not taking their medication as ordered by the doctor. (du Plessis et al. 2021:23)

Tesch’s method for data analysis was used. The results from the minor dissertation outlined the following three themes: firstly, ‘Family members’ experienced psycho-social effects of adult females living with depression who are non-compliant to psychiatric medication’; secondly, ‘Family members experienced treatment refusal by adult females living with depression who were non-compliant to psychiatric medication’ and thirdly, ‘Family members experienced challenges caring for adult females living with depression who were non-compliant to psychiatric medication’ (du Plessis et al. 2021:1).

Prior to this research, there was a dearth of understanding regarding the challenges these individuals face and the effectiveness of strategies to address their difficulties (Hunter, McCallum & Howes 2019:2). Therefore, the researcher asserts that this design greatly facilitated the comprehension of participants’ real-life experiences (Gray, Grove & Sutherland 2016:747). The chosen research design enabled the researcher to observe, describe and document the various aspects of the study as they naturally unfolded over three distinct phases (Polit & Beck 2018:226).

### Phase 1: Empirical phase

This phase encompassed two stages, namely systematic review and qualitative research methods.

#### Stage 1: Systematic review

A comprehensive systematic review was conducted to examine the prevalence of medication non-compliance among adults living with depression. This review followed a structured approach consisting of five key steps: formulating a clear research question, collecting and categorising data, critically evaluating the quality of the studies, summarising the findings and engaging in a thorough discussion of the evidence. To gather relevant studies, searches were conducted in reputable databases between 03 January 2022 and 30 June 2022 in databases such as Google Scholar, ScienceDirect and EBSCOhost. The critical appraisal process was then employed to assess the validity, reliability and applicability of the information obtained from these sources. Furthermore, the results of the review were subjected to scrutiny by an independent reviewer to ensure accuracy and reliability. This rigorous methodology allowed for a comprehensive analysis of the existing literature on medication non-compliance among adults living with depression.

**Results of systematic review:** The systematic review yielded significant results, highlighting three key themes. Firstly, the adults living with depression who are non-compliant with medication experience psychological challenges (forgetfulness, lack of awareness regarding the importance of medication and fear of side effects of the medication). Secondly, the adults coping mechanisms included substance abuse and lack of locus control. Thirdly, the adults who experienced social challenges (stigma and lack of social support) were identified as barriers to medication compliance in adults living with depression.

#### Stage 2: Qualitative research method

Ten in-depth phenomenological interviews were conducted, with an equal distribution of five males and five females. Open-ended questions were employed during these interviews to allow for in-depth exploration of the topic. These interviews were followed by a thorough review of previous results from the researchers’ minor dissertation to promote cross-validation.

### Data collection

The ward manager and independent fieldworker recruited participants. To ensure impartiality, the researcher employed an independent fieldworker during the recruitment process. The ward manager of the public hospital’s psychiatric ward identified prospective participants (adults living with depression who were non-compliant with their psychiatric medication) admitted to the psychiatric ward. The ward manager contacted the adults living with depression who were non-compliant and invited them to take part in the study. After the adults living with depression agreed to participate in the study, their contact information was provided to the fieldworker on a confidential basis. The fieldworker met the adults living with depression to arrange interviews with them and thoroughly explained the purpose, risks and benefits of the study to potential participants. A field worker is skilled in qualitative data collection and has signed a confidential agreement with the researcher before entering the field. Data were collected in the psychiatric ward of the tertiary academic hospital in one of the psychologist’s room to ensure privacy where adults living with depression who are non-compliant with medication are admitted. Data were collected through in-depth phenomenological interviews, employing a technique known as bracketing, setting aside any preconceived notions and focussing solely on the specific phenomenon being investigated. These interviews were kept concise, lasting no longer than 45 min. Data were collected until data saturation was achieved (Saunders et al. 2018:1895), and this was achieved on the tenth interview. To maintain accuracy and transparency, all interviews were audio-recorded with the prior consent of the participants, who willingly signed voluntary consent forms. Additionally, the field worker used pseudonyms to protect the identities of the participants.

### Research instrument

To gain an understanding of adults living with depression’s obstacles and needs, the independent field worker employed semi-structured interviews as a research instrument. These interviews were conducted using open-ended questions, allowing for a more in-depth exploration of the subject matter. In particular, the field worker directed the following inquiry towards adults living with depression who exhibited non-compliance with their medication: ‘How is it for you not taking your medication?’

### Population and sampling

A population is the entire group of individuals of interest to the researcher (Creswell & Creswell 2018:150). The accessible population in this study was adults living with depression who were non-compliant with medication. The study population consisted of 10 adults living with depression who were non-compliant with medication and who were admitted to the psychiatric ward of the tertiary academic hospital. Adults living with depression were purposively sampled (Moser & Korstjens 2018:121). A method for diagnosing medication non-compliance was used, which involved directly questioning patients about any difficulties they may encounter in following their medication regimen. This diagnostic approach relies on self-report assessments, assuming that patients’ responses are both truthful and accurate (Rusu 2023:239). The inclusion criteria for this study were adult males and females diagnosed with depression by a registered psychiatrist, using the Diagnostic and Statistical Manual of Mental Disorders, 5th edition (DSM-5). Additionally, participants needed to be adults between the ages of 18 and 65 who were non-compliant with their depression medication. They also needed to be able to communicate in English and be willing to participate in the research study. Finally, participants needed to have been admitted to a psychiatric hospital and diagnosed with depression by a registered psychiatrist. The exclusion criteria were male and female individuals younger than 18 years as well as adults living with other mental illnesses, except depression.

### Setting

The setting of this study encompassed both the psychiatric and mental healthcare wards within a level three tertiary academic hospital providing specialised medical and mental health services to patients in need. The tertiary academic hospital has allocated 18 beds to the mental healthcare ward, which caters to adults living with depression. This bed allocation includes nine male beds, seven female beds and two unigender beds.

### Data analysis

Data were analysed using content analysis. This method involves reducing data through the process of ‘coding’, which breaks down the data into smaller units. These smaller units are then organised into categories and themes (Aveyard & Goodman 2017:405).

To begin with, the audio-recorded interviews were transcribed verbatim by the researcher. Next, a coding scheme was developed to effectively code the data. Conceptual categories were created, assigning labels to important emerging concepts. Subsequently, the data were coded into themes and subthemes. Finally, the data were organised in a cohesive manner.

In order to ensure the reliability and validity of the analysis, a co-coder independently analysed the transcribed data and developed their own themes and subthemes. Following this independent analysis, a meeting was held between the researcher and the coder to discuss their findings. In cases where there were disagreements regarding themes and subthemes, a thorough discussion ensued to reach a consensus on the final themes and subthemes.

By employing this rigorous and systematic approach to data analysis, the researcher was able to ensure the accuracy and comprehensiveness of the findings. This method allowed the researcher and the coder to effectively analyse the data and draw meaningful conclusions.

### Trustworthiness

Credibility, dependability, confirmability, transferability and authenticity are essential in conducting a rigorous and reliable study (Polit & Beck 2018:295). Credibility was enhanced through employing two distinct data collection methods: a systematic review and semi-structured individual interviews. By using these approaches, the researcher aimed to bolster the believability of the findings, thereby establishing credibility with external readers. Dependability was achieved through a meticulous description and application of the research methodology. Additionally, an audit trail was implemented, triangulation was ensured, and a co-coder was selected to verify the findings. Confirmability was enhanced by diligently ensuring that the data accurately represented the information provided by the participants, as well as the results obtained from the systematic review. Transferability was attained through providing a comprehensive and detailed description of the research methodology, enabling readers to evaluate its applicability in various contexts. Authenticity was promoted by capturing the participants’ experiences, emotions, language and context; the researcher ensured that readers could genuinely understand and connect with the lives being portrayed.

### Ethical considerations

Ethical approval to conduct the study was obtained from the University of Johannesburg’s Research Ethics Committee (reference no. REC-673-2020), the University of Pretoria’s Ethics Committee (reference no. 737/2020) and Steve Biko Academic Hospital (reference no. GP_202102_021). Participants’ rights were protected; ethical principles were identified and adhered to throughout the study. Four principles were considered and adhered to when conducting research, namely autonomy, non-maleficence, beneficence and justice. In this study, all these principles were adhered to throughout the research process.

The authors ensured the right to anonymity and confidentiality. Participation in the study was voluntary, and withdrawal was welcomed with no penalties. Written consent was obtained from all participants. Collected data were only accessible to authors and the independent coder.

## Discussion of results

The central concept in this study was obtained after merging three key data sources. Firstly, the systemic review conducted over a period of 6 months. Secondly, the results of a minor dissertation titled ‘Family members’ lived experiences of non-compliance to psychiatric medication given to female adults living with depression’ (du Plessis et al. 2021:4) were considered. Thirdly, the findings of in-depth phenomenological interviews conducted with adults living with depression. These three sets of results were merged, and a cross-validation report was conducted. Cross-validation is a widely recognised qualitative research validation strategy closely linked to triangulation. The cross-validation analysis revealed that adults living with depression and their family members shared similar experiences regarding medication non-compliance. Adults living with depression were unaware of how their disruptive behaviour impacted their families. Similarly, families were unaware of the struggles their relatives living with depression faced, including the side effects of medication and severe depressive moods, which contributed to their lack of motivation to comply with treatment regimens.

Theme one indicated that intrapersonal challenges and non-compliance resulted in emotional turmoil. According to Yager (2021:616), interpersonal challenges can be attributed to the pain or anguish experienced by adults living with depression and family members caring for those who are non-compliant with their medication. Non-compliance also has an impact on their relationships with others. Asadollahi et al. (2021:7) indicate that non-compliance with medication among adults living with depression can lead to various negative emotions, including anxiety, depression, anger and despair. This can result in a loss of control and emotional turmoil, which can be particularly challenging for family members caring for adults living with depression.

One of life’s most frustrating and emotionally straining experiences for family members is caring for an adult living with depression who is non-compliant with their medication. Anxiety, helplessness, guilt, sorrow, confusion and other negative emotions are difficult to manage when caring for an individual with depression (September & Beytell 2019:1163). The most common reaction by family members caring for these adults is resentment and anger (Black 2020:n.p.), and it is natural for the family members to be overwhelmed by the heavy load of caring for these relatives. Blank (2020:143) therefore argues that it is vital to communicate openly and keep lines of communication open to build trust and find the best strategies to address the challenges individuals face constructively.

Theme two reflected that non-compliance led to adults living with depression and their families resorting to various coping mechanisms that relied on an external locus of control. Instead of assuming responsibility for their actions and the resulting consequences, adults living with depression often rely on external forces, such as luck, fate, or other people to rescue them or address the issues they face. This indicates their inclination to seek solutions from external factors rather than taking ownership of their actions and the outcomes. This approach can be detrimental to personal growth and development, as it limits individuals’ ability to take charge of their own lives and make positive changes, reflecting an internal locus of control.

In this study, the adults living with depression and family members coped with medication non-compliance in several ways. These coping mechanisms included withdrawal and isolation, aggression and being inaccessible in their direct engagement with others, giving up on eating, being unreasonable, attempting to control the individual by laying down rules, developing routines, setting boundaries and avoiding them altogether. While these coping mechanisms may seem like a solution, they can exacerbate the problem and create more tension within the family dynamic. Adults living with depression and family members must ultimately approach medication non-compliance with empathy and understanding while collaborating to find a solution that benefits everyone involved (Wang et al. 2022:67).

When adults living with depression fail to comply with their medication regimen, it is frustrating and concerning for those around them (Chand, Arif & Kutlenios 2021:n.p.). However, it was essential to recognise that underlying causes for this behaviour may exist. The medication could be causing unpleasant side effects, and the adult living with depression might be struggling with the cost of the medication. By approaching the situation with empathy and understanding, family members can create a safe and supportive environment for these individuals to express their concerns (Stroup & Gray 2018:341). This can lead to a more productive conversation and solutions for everyone involved. It is also essential to involve healthcare professionals in the discussion. They can provide valuable insight into alternative medications or treatment options that may suit the afflicted individuals (Sarkhel, Singh & Arora 2020:319).

Theme three indicated that for adults living with depression who are non-compliant with medication, interpersonal challenges result in a lack of support and isolation. Adults living with depression can display negative behaviours, such as impulsive and potentially disruptive actions and erratic verbal outbursts, or they may not comply with the rules and expectations of those around them (Fariba & Gokarakonda 2020:n.p.). Such behaviours make it challenging for the affected adults and their families to participate in social activities, where they may be seen as disruptive, or they may refuse to engage in a specific activity because of their lack of compliance. This leaves the family feeling isolated and without any support from those around them.

The challenge of managing such behaviours is further compounded when the adult living with depression behaviours becomes unpredictable, leaving those around them unsure of what to expect or unable to make plans based on this behaviour (Blumenthal, Wood & Williams 2018:n.p.). This can result in isolation, frustration and anxiety for the adult living with depression and their family. By providing space to build healthy relationships and trust, and by offering an outlet for the affected individuals to express themselves, families can start to take steps to alleviate the interpersonal challenges that non-compliance with psychiatric medication creates (Tong et al. 2020:174).

### Phase 2: Classification of the central concept

The aim of the study was to develop a comprehensive conceptual framework for psychiatric nurses to effectively facilitate medication compliance among adults living with depression, a group that often faces significant barriers to compliance because of the complex interplay of psychological, social and pharmacological factors. The framework is designed to be a guiding tool for psychiatric nurses to understand the multifaceted nature of depression and its impact on patient’s willingness and ability to follow prescribed medication regimens.

The researcher classified concepts to develop a conceptual framework. During concept classification, the results of the empirical phase (systematic review, family members’ lived experiences of non-compliance to psychiatric medication given to female adults living with depression and experiences of adults living with depression who were non-compliant with their psychiatric medication) were used during concept classification. As previously described, the results of family members revealed three themes:

[*E*]xperienced psycho-social effects of adult females living with depression who were non-compliant to psychiatric medication, experienced treatment refusal by adult females living with depression who were non-compliant to psychiatric medication and experienced challenges caring for adult females living with depression who were non-compliant to psychiatric medication.

The results of the adults living with depression revealed two themes:

[*A*]dults living with depression are non-compliant with medication for various reasons and adults living with depression agree that not taking medication as prescribed creates a setback to their mental health recovery.

The results of the systematic review revealed three themes:

[*E*]xperienced psychological challenges [*forgetfulness, lack of awareness regarding the importance of medication and fear of side effects of the medication*], coping mechanisms included substance abuse and lack of locus control and lastly, experienced social challenges [*stigma an d lack of social support*] were identified as barriers to medication compliance in adults living with depression.

**FIGURE 1:**
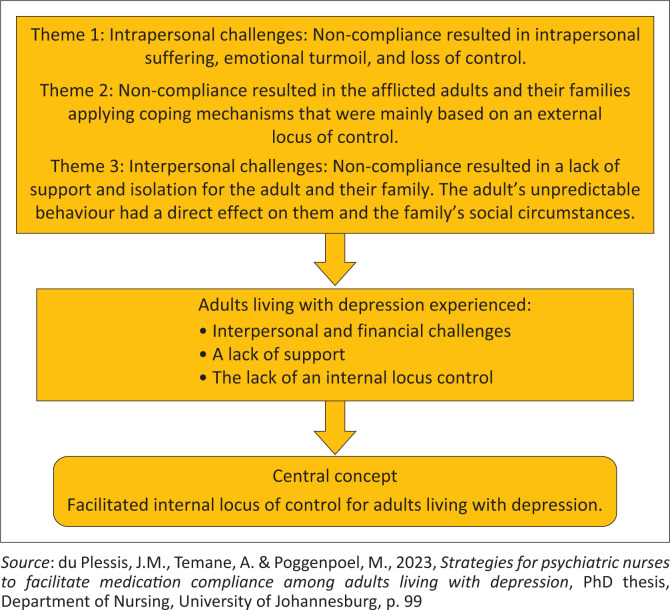
Central concepts to facilitate adults living with depression’s internal locus of control.

Figure 1 visually depicts the central concepts in the conceptual framework. This illustration serves as a valuable tool for understanding the key components of the framework and their interrelationships.

Based on the identified central concepts, the researcher developed a thinking map according to Dickoff, James and Wiedenbach’s (1968:415) survey list. The thinking map directed the development of the conceptual framework, as discussed in this section.

The thinking map presents the structure of the concepts and interactions between the agent and the recipients. The procedure and process of the activity, the context, dynamics and outcome are described and discussed to construct the conceptual framework. The interaction and dynamics between the agent and the recipients are also contextualised in a specific framework and procedure. The recipients’ roles and activities are outlined to attain the desired outcome via the facilitation process to ensure the facilitation of an internal locus of control for adults living with depression who are non-compliant with their medication.

Aspects from the survey list are included and described next to ensure they are understood in the context of this study, as illustrated in Table 1.

**TABLE 1:** Thinking map.

Primary agent Secondary agent	Advanced psychiatric nurse The psychiatric nurse
Primary recipient Secondary recipient	Adults living with depression Family members
Procedure	Facilitation of internal locus of control for adults living with depression
Dynamics	Interpersonal and financial challenges Lack of support Lack of internal locus control
Context	Mental health ward Level 3 academic hospital
Outcome	Facilitated internal locus of control for adults living with depression

*Source*: Dickoff, J., James, P. & Wiedenbach, E., 1968, ‘Theory in a practice discipline, part 1: Practice oriented theory’, *Nursing Research* 17(5), 415–435. https://doi.org/10.1097/00006199-196809000-00006

### Phase 3: Development of the conceptual framework to facilitate medication compliance among adults living with depression using an internal locus of control

**FIGURE 2:**
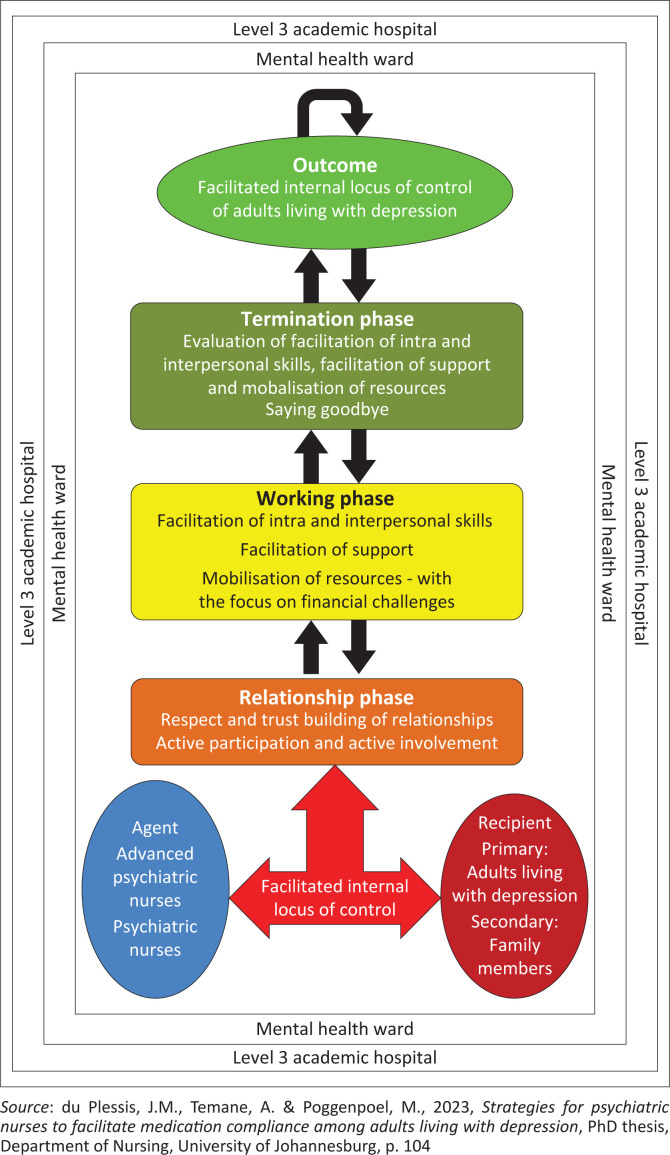
Conceptual framework to facilitate adults living with depression’s internal locus of control.

Once the concepts in Figure 1 were classified, a conceptual framework was developed to enhance medication compliance among adults living with depression using an internal locus of control. This framework was then finalised and mapped out, as shown in Figure 2.

### Description of the conceptual framework

The conceptual framework provides a schematic picture of the inter-relationships among the different concepts and should reflect researchers’ perspectives of their findings, as stated by Polit (2021:549). The conceptual framework positions the researcher in the study. It includes all relevant concepts that were considered, defined, integrated and discussed. The conceptual framework to facilitate adults living with depression’s internal locus of control is presented in Figure 2. The blue colour on the left-hand side is the agent, the agent is the first process of the framework. A dark red colour represents the recipient. The colour white is chosen for the context of this conceptual framework. The procedure consists of three phases, namely: The relationship phase represented by an orange colour, the working phase represented by the colour yellow and the termination phase shown by an olive colour. The outcome is represented by a green colour. All shapes on the conceptual framework are connected to each other with arrows that represent relationships between these processes.

### Components of the conceptual framework

#### Agent

Agents are responsible for performing the activity (Dickoff et al. 1968:423). The agent is primarily the advanced psychiatric nurse who provides support for psychiatric nurses to facilitate adults living with depression to develop an internal locus of control. The advanced psychiatric nurse is a skilled therapeutic practitioner who facilitates medication compliance by mobilising resources for psychiatric patients. The advanced psychiatric nurse thus establishes a trusting relationship with adults living with depression to facilitate their internal locus of control.

#### Recipient

Recipients refer to the who or what that receives the activity to be performed (Dickoff et al. 1968:423). As indicated earlier in this article, the primary recipients are adults living with depression, and their families are secondary recipients. The family is responsible for supporting the adults living with depression while facilitating their internal locus of control.

#### Context

The context refers to the setting where the framework will be implemented (Dickoff et al. 1968:423). The context of this study is a mental health ward where adults living with depression are admitted to promote their mental health. The advanced psychiatric nurse will guide the process of facilitating their internal locus of control within the context of the mental health ward. Context is a prerequisite for understanding experiences or a particular phenomenon and can be described as the circumstances that form the setting of an event, statement or idea (Cooper, Heron & Heward 2020:n.p.).

### Procedures: Facilitation of internal locus of control

The question to address in terms of procedures is: what are the guiding procedures, techniques or protocols to implement this framework (Dickoff et al. 1968:423)? Facilitation is a crucial process used by trainers, team builders, meeting leaders, managers and communicators to enhance a group or team’s content, process and structure (Simonson 2019:n.p.). In addition, facilitation involves making an action or process more accessible and manageable for adults living with depression, ultimately leading to a more productive and efficient outcome. According to the Collins English Dictionary (2018:n.p.), facilitation makes something possible or more manageable. In essence, facilitation is the art of guiding a group towards a common goal while meeting each individual’s needs.

Facilitation is a process that advanced psychiatric nurses can use to assist adults living with depression in developing an internal locus of control to cope effectively with the challenges related to their non-compliance with medication. To effectively facilitate, advanced psychiatric nurses must maintain objectivity and use facilitative communication skills such as listening, empathy, reflection, summarising and paraphrasing to assist adults living with depression in complying with their medication. Therefore, during group processes, the advanced psychiatric nurse must remain neutral and unbiased (Hartley et al. 2020:103490).

#### Relationship phase

The relationship phase is the starting point in the facilitation process, where the groundwork and orientation occur, and relationships are established between the advanced psychiatric nurse and the adults living with depression. Therapeutic tasks that need to be accomplished by the advanced psychiatric nurse during the relationship phase include respecting and building trusting relationships, and active participation and involvement (Scheydt & Hegedüs 2020:5).

The advanced psychiatric nurse will create a trusting relationship with the adults living with depression. The advanced psychiatric nurse and adults living with depression will get to know each other face-to-face to establish relationships and encourage group cohesion. Adults living with depression will be oriented about the facilitation processes and protocol to follow (Colligan et al. 2020:341). Mutual respect and trust are encouraged in establishing group guidelines. During the relationship phase, the advanced psychiatric nurse will endeavour to impart and create a relationship of trust among the adults living with depression, as suggested by Fieldhouse (2022:30). Trust is the initial developmental task (Ozaras & Abaan 2018:629), and if it is not achieved in the relationship phase, the advanced psychiatric nurse will find it challenging to facilitate adults living with depression’s internal locus of control.

The advanced psychiatric nurse will endeavour to foster active participation and involvement from the adults living with depression in achieving the outcome of the facilitation of internal locus of control. Demonstrating a sincere interest in adults living with depression can prove beneficial during this information-gathering phase. Active listening and empathy are also vital in encouraging active participation and involvement from adults living with depression. Active listening is a critical skill for advanced psychiatric nurses to possess when working with adults living with depression (Bletscher & Lee 2021:151). Moreover, empathy is a crucial component of effective communication and therapeutic relationships (Moudatsou et al. 2020:2). It allows individuals to connect with others on a deeper level, fostering trust and understanding. By accurately perceiving and acknowledging the adults living with depression’s emotions, the advanced psychiatric nurse can provide support and assistance in a meaningful and beneficial way.

#### Working phase

In the working phase, the advanced psychiatric nurses will use their skills to facilitate intra and interpersonal skills, and support and mobilise resources for adults living with depression. During the working phase, process activation takes place. The adults living with depression can converse and openly share their experiences of medication non-compliance with the advanced psychiatric nurse. The facilitation processes in the working phase will assist the advanced psychiatric nurse in achieving the study’s intended outcome.

**Facilitation of intra and interpersonal skills:** Interpersonal skills refer to the ability to communicate and interact effectively with others. In contrast, intrapersonal skills involve self-awareness and self-management (Wallender et al. 2020:86). Facilitating interpersonal skills is about putting adults living with depression in direct control to communicate and interact proficiently with others. These skills are essential in personal and professional settings, enabling adults living with depression to build relationships, collaborate effectively and resolve conflicts efficiently. Listening actively, expressing oneself clearly and empathising with others are all crucial components of interpersonal skills (Brown, Yu & Etherington 2020:188).

The advanced psychiatric nurse will use the following approaches to promote intra and interpersonal skills, and the focus is on enabling adults living with depression to cope with the challenges of non-compliance with medication. With intrapersonal skills, the advanced psychiatric nurse should focus on self-awareness, self-management, mindfulness, adaptability and resilience. To promote the facilitation of interpersonal skills, advanced psychiatric nurses should focus on basic communication skills such as active listening, building relationships and managing conflict.

Furthermore, the management of conflicts is critical to promote effective interpersonal communication. It requires an understanding of all sides of the conflict and seeking mutually acceptable solutions. In this regard, effective communication skills, including active listening and empathy, are vital for handling conflicts with respect and understanding (Ronquillo, Ellis & Toney-Butler 2023:n.p.)

For adults living with depression, interpersonal skills such as self-awareness, self-management, mindfulness, adaptability, resilience, active listening, relationship building and conflict management are imperative to communicate effectively (Matveyeva 2023:n.p.).

**Facilitation of support:** Adults living with depression need assistance to cope with medication non-compliance. Based on the study’s findings, it was clear these individuals experienced a lack of support. This study confirmed these individuals also had to deal with a lack of available information regarding depression and medication non-compliance. The facilitation of support will thus involve the following activities: the facilitation of support groups, and the facilitation of empowerment through education on depression.

Adults living with depression often face significant challenges and require a supportive environment to help them cope. A support group can provide a safe space for these individuals to share their experiences and common problems associated with their condition. A support group gathers individuals who share a particular problem, condition, illness or personal circumstance (Worrall et al. 2018:n.p.).

The support group for adults living with depression is meant to provide a non-judgemental and caring atmosphere where individuals can mutually support one another by sharing their thoughts, ideas, concerns, questions and coping strategies (Jordan 2023:n.p.). Such a support group is essential in promoting comprehensive mental healthcare (Singh, Kumar & Gupta 2022:n.p.). It provides group members with an opportunity to connect with others going through similar experiences and can offer practical awareness from personal experience (Grundman, Edri & Stanger Elran 2021:26).

Adults living with depression must have access to a safe and supportive environment where they can work through the challenges of their condition (Mayo Clinic 2023:n.p.). Support groups offer a unique opportunity for these individuals to exchange information, share practical tips and strategies and express their feelings without fear of judgement (Rashid & Seligman 2018:n.p.). By participating in a support group, adults living with depression can find solace in their experiences being typical and expected (Gosling, Parry & Stamou 2021:21), which can help alleviate the feelings of isolation and loneliness often associated with depression.

**Mobilisation of resources with a focus on financial management:** Adults living with depression often encounter difficulties in managing their finances. Coping with the condition can be financially challenging, and practical financial management strategies can be crucial in reducing the burden and strain of this mental health condition (Ryu & Fan 2022:18). Empowering afflicted individuals with financial management skills is essential, and this can be achieved through a structured approach. The facilitator should differentiate between needs, expenses and wants, and provide guidance on saving money and creating a budget. By adopting a proactive approach to financial management, adults living with depression can improve their overall well-being and reduce their condition’s negative impact on their financial stability (Essel-Gaisey et al. 2023:2).

### Termination phase

The Latin *terminus* refers to the ending of an action as an indicator that the activity’s goal has been met (Oxford Learners Dictionaries 2023:n.p.). This framework’s termination phase will focus on assessing adults living with depression who cultivated an internal locus of control to promote medication compliance. Termination provides an opportunity to evaluate relationships intentionally and meaningfully and encourages a continuation of personal growth for each person (Uggla 2019:233).

The advanced psychiatric nurse will prepare the adults living with depression for the termination phase in advance. During the termination phase, adults living with depression will be invited to share experiences of the facilitation of internal locus of control. The advanced psychiatric nurse will assess their knowledge, skills and attitudes related to their facilitated intra and interpersonal skills, support and mobilisation of resources. When adults living with depression possess knowledge, skills and attitudes that reflect the crucial significance of intra and interpersonal skills, support and resource mobilisation, they are able to foster an internal locus of control to enhance continuous medication compliance. Adults living with depression often want to perform more appropriately and are encouraged to experience an internal locus of control as meaningful and purposeful in their everyday lives to promote their mental health (Francis 2014:1369). The advanced psychiatric nurse and the adults living with depression will say goodbye and wish each other well.

### Outcome

The outcome refers to the end of the final achievement of the activity, reflecting that the goal was achieved. The goal is to help adults living with depression achieve an internal locus of control by enhancing their intra and interpersonal skills, providing support and mobilising resources (Johanna Elisa Dietl et al. 2023:2). When this outcome has been achieved, it will indicate that adults living with depression are ready to be responsible for their own mental health by complying with medication.

### Relevancy of the conceptual framework

This conceptual framework holds great significance in guiding psychiatric nurses as they work towards empowering adults living with depression to develop an internal locus of control. It emphasises the importance of addressing any unresolved issues in order to effectively guide adults living with depression. Based on this premise, this conceptual framework assumes that each concept within it will add value to the desired outcome of promoting an internal locus of control among adults living with depression.

### Limitations

Obtaining approval for research involving human subjects requires meticulous coordination and a thorough review by relevant institutions. This often results in time constraints that impact the timely commencement and completion of research, as was the case in this study. The ongoing coronavirus disease 2019 (COVID-19) pandemic further exacerbated these challenges, particularly regarding data collection.

With lockdown measures and social distancing guidelines in place, traditional data collection methods, such as face-to-face interviews, became impractical and consequently led to this study’s temporary pauses for brief periods at times. In addition, only one mental health unit was used for this study. To effectively support families affected by depression, it is imperative to extend understanding beyond individual adults and consider the collective mental well-being of the entire family unit. It is thus crucial to provide comprehensive support and address the broader impact of depression on families as a whole.

### Recommendations

The recommendations stemming from this study are categorised under recommendations for mental health practice, nursing education and nursing research.

#### Recommendations for mental health practice

From the research findings, it is evident that adults living with depression are burdened by an external locus of control instead of relying on their internal locus of control to comply with their medication. Mental health practice should be tailored specifically for adults living with depression and their families to strengthen their coping skills and support systems. A programme for families and adults living with depression should also be initiated in mental health practice to provide them with essential coping mechanisms to effectively address non-compliance with medication.

#### Recommendations for nursing education

It is recommended that medication compliance be integrated into the psychiatric nursing curriculum. This will equip student nurses with the necessary knowledge and skills to effectively assist adults living with depression in complying with their medication.

The development of an in-service training programme on medication compliance for adults living with depression plays a vital role in enhancing nurses’ professional development. It will be a valuable opportunity for them to acquire essential skills and knowledge. Moreover, by actively participating in in-service training programmes, nurses can enhance their expertise and stay abreast of the latest advancements in the psychiatric field. This benefits the individual nurses and contributes to the overall quality of patient care.

#### Recommendations for nursing research

The reviewed literature shows no existing conceptual framework currently addresses the use of an internal locus of control to promote medication compliance in Gauteng province. The researcher thus needed to develop a conceptual framework for psychiatric nurses to facilitate adults living with depression’s internal locus of control to promote medication compliance. Further research could explore psychiatric nurses’ implementation of the conceptual framework to enhance medication compliance among adults living with depression.

Furthermore, there is a need for more research on the effectiveness of medication initiation and participative programmes in diverse settings. By investigating the outcomes of such programmes, psychiatric nurses can gain valuable insights into their efficacy and adaptability, ultimately enhancing patient outcomes.

## Conclusion

The purpose of this study was to develop a conceptual framework aimed at enhancing adults who are living with depression’s internal locus of control. A qualitative, exploratory, descriptive and contextual research design was thus employed. The study followed a systematic approach, which involved thoroughly reviewing existing literature, classifying relevant concepts and ultimately developing a conceptual framework. Dickoff et al. (1968:422) outlined six steps to guide this development. The resulting framework not only provides valuable insights but also serves as a valuable tool for future research endeavours aimed at enhancing medication compliance among adults living with depression.
